# Supplementary material for: Are carbon emissions trading and green financial instruments synergistic? -Comprehensive quantitative research based on content analysis
Source: PLoS One. 2024 Mar 7;19(3):e0298601. doi: 10.1371/journal.pone.0298601 (PMC10919601; doi:10.1371/journal.pone.0298601)
Supplement: S1 Data — (ZIP) [file pone.0298601.s001.zip › Data/地级市Z_F工作报告.zip!/░▓╗╒/╦▐╓▌╩╨/└┤╫╘╣1⁄2╓┌║┼í╛┬φ┐╦╩2╛▌═°í┐_2007.docx]

2007年2月9日宿州市第二届人民代表大会第四次会议

代市长  吴旭军

各位代表：

现在，我代表市人民政府，向大会报告工作，请予审议，并请市政协委员和其他列席人员提出意见。

一、团结奋进，“十一五”发展开局良好

2006年，是“十一五”规划的开局之年。市政府在市委的正确领导下，团结带领全市人民，坚持以科学发展观为统领，深入贯彻党的十六届六中全会、省第八次党代会和市第三次党代会精神，抢抓机遇，攻坚克难，圆满完成了市二届人大三次会议确定的主要目标任务。全市生产总值完成359亿元，增长11.5%；财政收入16.8亿元，增长25.7%；城镇居民人均可支配收入8945元，农民人均纯收入2495元，分别增长13.1%、15.7%。各项社会事业协调发展，在新的起点上实现了新的跨越。

农村经济全面发展。农业生产喜获丰收，粮食总产达到368万吨，创历史最好水平。“双百工程”深入实施，大宗农产品优质率大幅提升，畜禽规模化养殖快速发展。产业化经营取得新突破，新增省市级龙头企业34家，农民合作经济组织发展到376个。农产品质量安全和标准化建设得到加强，埇桥区获得国家绿色农产品生产示范基地认证。“阳光工程”成效明显，累计培训农民工5.6万人，劳务输出总量突破120万人。新农村建设全面启动，“十镇百村”示范工程有序推进。“村村通”工程建成1446公里，节水灌溉和重点水利工程建设继续加强，万里绿色长廊二期建设、粮食产业化、农机化综合水平居全省前列。

工业带动持续增强。大力实施工业三年倍增计划和全民创业行动，工业增速逐季攀升。规模以上工业增加值完成55.5亿元，增长26.7%。实施皖北药业、雪龙化纤、安特生化、润达纺织、新锦丰食品、惠丰电源等千万元以上企业技改和扩建项目85个，完成投资42.5亿元，增加规模企业52户、产值超亿元企业10家；县区规模以上工业增速均超全市平均水平，增幅创近六年新高。民营经济快速发展，新增私营企业782家、个体工商户9082户。工业化、城镇化的推进，带动了服务业的快速发展，全社会消费品零售总额实现97亿元，增长13.6%。二、三产业对经济增长的贡献率达76%，成为推动经济增长的主导力量。

项目支撑更加有力。坚定不移地实施项目带动战略，着力提升“6+3”工程，固定资产投资增势强劲，年度投资规模突破百亿元，达到109.3亿元，增长28.6%。重大项目建设进展顺利，奎濉河干流治理基本完工；华电一期两台机组年内将实现投产，博洋纺织、电光防爆电气、虹光粮食深加工等项目加快建设；丰原60万吨农产品加工、金黄庄煤矿等项目正式开工；华电国际秸秆发电、泗永高速公路、钱营孜煤矿等项目即将开工；华电二期完成了所有支持性文件，将参加工程评优，林浆纸一体化已落实投资主体，明徐高速、宿淮铁路列入苏皖交通合作协议，形成了重大项目相继开工、滚动发展的良好态势。

对外开放深入推进。全面加快东向发展，与徐州市开展了党政代表团互访，在交通对接、旅游开发等方面进行深度合作，融入徐州经济圈取得新的进展。招商引资成效显著，全年落地300万元以上项目673个，实际到位内资62.5亿元、外资3250万美元，分别增长40%、19.5%。第三届中国宿州黄淮海地区农资交易会和第二届中国宿州灵璧石文化节成功举办，《走进宿州》专题片在央视播出，宿州的知名度和美誉度进一步提升。开发区基础设施和项目建设加快推进，集聚效应日益凸显。对外贸易持续增长，进出口总额完成4136万美元，按可比口径增长9%。

城市形象显著提升。加快皖东北中心城市建设，按照“一疏散、两集中”的总体要求，基本编制完成新一轮城市五年建设规划和宿城教育布局调整、城市绿地、供热、人防等专项规划。政务中心投入使用，汴河新区规划开始启动。城市基础设施建设完成投资6亿元，迎宾大道、淮海南路等12项工程先后竣工，雪枫公园、银河公园等12项工程正在加紧施工，市政公用设施更加完备，城市服务功能明显增强。综合开发势头强劲，全年竣工房地产面积116万平方米。城市管理体制不断完善，初步构建了市、区、街道三级管理，社区参与的城市管理新格局。实施“洁净、美化、亮化、畅通”工程，城市脏、乱、差得到有效治理。各县县城建设步伐加快，泗县东城新区、萧县凤山新区、砀山政务新区、灵璧滨河新区建设陆续启动，旧城改造全面推进。朱仙庄、黄楼、圣泉、下楼、黄圩等一批乡镇工业功能区建设初具规模，城镇化率提高3个百分点。

社会事业长足进步。科技创新步伐加快，国家农业科技入户工程试点顺利实施，科技创业服务中心投入运行。“以县为主”的农村义务教育体制改革逐步完善，普通高考本科上线人数连续15年居全省前列，10.4万名学生领用免费教科书，13万平方米中小学危房得到改造。市、县两级疾病预防控制和医疗救治体系基本建成，县、乡、村三级卫生服务网络进一步健全。文化事业加快发展，泗州戏和萧县农民画、灵璧钟馗画等7个项目分别入选国家、省级非物质文化遗产名录，埇桥区被命名为“中国马戏之乡”。竞技体育成绩显著，在省十一届运动会上获得金牌51枚，杨明明获第三届世界杯武术散打比赛冠军。国土资源、计划生育、环境保护等基本国策得到较好落实，土地开发复垦整理超额完成任务，重大项目建设用地基本保障；低生育水平继续稳定，出生人口性别比稳步下降；生态市建设力度加大，埇桥、萧县被批准为省级生态综合示范区。群众性精神文明创建活动扎实开展，公民道德素质和文明程度进一步提高。老龄、妇女、儿童及残疾人的合法权益得到保障。“五五”普法全面启动。国防动员、民兵预备役、人防建设和“双拥”工作进一步加强。人事编制、民族宗教、外事侨务、广播电视、新闻出版、防震减灾、物价、统计、档案、外宣、对台、红十字会、地方志等工作也取得新进展。

群众生活日益改善。就业和社会保障体系进一步完善，城镇新增就业岗位2.3万个，城镇登记失业率控制在4.3%以内；养老、失业、医疗、工伤等新增扩面3.77万人，企业退休人员养老金人均月增179.7元，城市低保年度人均月补差65.8元。积极推进社会福利、扶贫开发事业发展，困难群众生活、五保对象供养得到改善。加强安全生产专项整治，重特大安全事故得到有效防范。扎实开展“两访户”集中整治和领导干部信访接待日活动，人民群众合理诉求得到较好解决。严厉打击各种刑事犯罪和经济犯罪，群众的安全感进一步增强。

政府职能加快转变。深入开展纪律作风集中整顿活动，切实加强“两风”建设，一批群众反映强烈的问题得到有效整治，广大干部的责任意识、规则意识明显增强。行政审批制度改革继续深化，行政服务中心功能逐步完善，政府工作效率和服务水平不断提高。自觉接受人大法律监督和政协民主监督，注重听取各方面的意见和建议，认真办理人大代表议案4件、建议75件、市政协委员提案166件。编制完成覆盖全市重点部门、重点区域的应急预案，政府保障公共安全的能力进一步提升。强化行政监察和审计监督，认真开展商业贿赂专项治理，切实纠正部门和行业不正之风，政府廉政建设进一步加强。

各位代表，过去的一年，面对国家实施宏观调控的新形势，我们不仅保持了经济稳健增长、社会协调发展的态势，营造了同舟共济、合力攻坚的氛围，夯实了持续发展、跨越发展的基础，而且进一步坚定了全面追赶的信心，增添了重点突破、局部超越的勇气。这些成绩的取得，主要得益于市委的正确领导和历届班子打下的坚实基础；得益于全市干部群众不断解放思想，开拓进取，始终不渝地把谋发展、快发展作为共同目标和不懈追求；得益于全社会各个方面，包括外来投资者积极参与、支持宿州的建设和发展。在此，我谨代表市人民政府，向全市广大工人、农民、知识分子和离退休老同志，向各民主党派和人民团体，向驻宿解放军、武警官兵和政法干警，向所有支持宿州发展、参与宿州建设的社会各界人士，致以崇高的敬意和衷心的感谢！

各位代表，过去的一年，发展成就令人振奋，但存在的问题也不容忽视。应当清醒地看到，我市在省内人口总量靠前、经济总量居中、人均水平偏后的局面尚未改变；工业化、城镇化和农业产业化水平较低的现状依然存在；固定资产投资不足，即期发展速度相对偏慢；社会事业和公共服务建设滞后，影响和谐稳定的因素较多；少数干部思想解放的程度不深，抓落实的力度不够，服务意识和办事效率有待进一步提高。对此，我们将以高度的责任感和使命感，坚持不懈地用改革和发展的办法加以解决。

二、把握形势，明确2007年发展目标

2007年是深入实施“十一五”规划，全面落实市第三次党代会战略部署的重要一年，也是实现工业倍增计划、创建省级文明城市和园林城市三年目标的关键一年。政府工作的总体要求是：认真贯彻市第三次党代会的战略部署，紧紧围绕“全面追赶、重点突破、局部超越”的发展要求，坚持以科学发展观为统领，以总量扩张、结构调整为主线，以工业化、城市化为核心，深入实施五大战略，着力解决影响经济发展和社会和谐的突出问题，加快实现“两个跃升、一个崛起”的奋斗目标。

主要预期目标是：全市生产总值增长11%左右，总量力争突破400亿元；财政收入保持同步增长，总量力争突破20亿元；全社会固定资产投资增长25%以上；城镇居民人均可支配收入、农民人均纯收入分别增长10%和7%；城镇登记失业率控制在4.5%以内；人口出生率控制在13‰以内。

确定上述目标，是努力缩小宿州与全国、全省平均水平差距的迫切需要，是解决当前经济社会生活中突出问题的客观要求。在实际工作中，我们要力争发展得更好更快，各县区应当确立更高的目标，不断开创经济社会发展的新局面。

实现上述目标，我们面临难得的发展机遇。国家将加大对直接改善农村生产生活条件和基础教育、公共卫生等社会事业的投入，继续巩固、完善、加强各项支农惠农政策，中部崛起战略进入操作层面，东向发展深入实施，资本流动和产业转移不断加速，外部发展环境总体较好。经过多年努力，我市奋力崛起的思路更加清晰，经济增长的内在动力逐步增强，特别是市第三次党代会胜利召开，市县乡三级党委换届圆满完成，全市上下人心思变、人和业兴的氛围更加浓郁，具备了快速发展的支撑条件。在工作实践中，我们必须坚定不移地推进思想解放和观念创新，把落实宏观调控政策与宿州实际结合起来，不断增强工作的前瞻性和创造性；必须坚定不移地加快发展，全面掀起大开放、大建设、大发展的热潮，努力巩固快速发展的良好势头；必须坚定不移地抓住东向发展、工业强市、项目支撑、中心城市带动、区域协调发展等战略重点，并不断深化、提升和完善；必须坚定不移地加快经济结构调整，尽快实现发展形态由农业为主向工业主导型转变，加速工业化、城镇化进程；必须坚定不移地促进社会和谐，坚持一手抓富民，一手抓安民，为奋力崛起创造更加和谐的社会环境。

各位代表，面对百舸争流、千帆竞发的区域经济发展态势，亦步亦趋只会拉大差距，跨越发展才能奋力崛起。我们坚信，只要全市上下凝心聚力，发奋图强，夺先发之机，激后发之势，就一定能够把宿州的发展大业推向前进！

三、乘势而上，推动经济又好又快发展

加快发展是宿州当前最紧迫的任务，我们必须坚持以“快”发展推动“好”发展，加快扩张经济总量，转变增长方式，提升发展质量，增强综合竞争力。

（一）强力推进工业三年倍增，快速扩张工业总量。把快速扩张工业总量作为经济工作的重中之重，按照“大项目、产业链，大基地、产业群”的思路，努力打造亿元项目、十亿元企业、百亿元产业，加快建设煤电能源、加工制造和农副产品深加工三个百亿元基地，形成一批支柱产业和“顶天立地”的领军企业。坚持扶优扶强，加大政策扶持和要素倾斜，推动现有企业加快内涵改造和外延扩张，支持优势企业上市发展。加强企业管理创新、技术创新和经营创新，新创一批省级和国家级名牌产品，增强核心竞争力。支持皖北煤电、中煤三建等驻宿企业做大“总部经济”，推进跨行业扩张。实施中小企业成长工程，壮大中小企业集群，今年再新增规模企业60户，总量突破300家。加快企业改革步伐，完成116户国有集体企业改制任务。

着力改善工业发展条件。集全市之力重视工业、支持工业，做到人才向工业汇聚，精力向工业集中，要素向工业倾斜，努力形成全市上下大办工业、办大工业的良好氛围。加强金融生态建设，积极搭建银企合作平台，加强银企项目对接和发展互动，加快徽商银行等金融机构的入驻，设立中小企业担保公司，支持农业发展银行商业化经营，切实解决企业融资难题。抓好城乡电网建设，确保能源供应。严格落实工业发展考核办法和奖惩措施，加强工业经济运行调节，抓好重点行业、重点企业监测，确保规模以上工业增加值增长25%以上。高度重视企业家和各类创业技术人才队伍建设，为工业发展提供人才支撑。

（二）加速中心城市建设，增强城市辐射力和带动力。把做大做强中心城市作为加快发展的战略支点，以创建省级文明城市和园林城市为目标，围绕拓展宿城规模，提升城市品位，推动城市建设由线到面，加快区域改造建设步伐。以西昌南路、纺织中路两侧连片开发为重点，实施西南片区建设；以拂晓大道、人民路向北延伸为依托，推进汴河新区建设；以淮河东路、浍水东路建设为突破口，启动道东片区改造，充实完善“L”型框架，实现三大片区联动发展。全面加快城市基础设施建设，逐步完善市政道路交通网络。加大城市绿化投入，抓好关键节点景观改造，形成全方位、立体式绿化格局，力争城区绿化率三年翻一番。继续加大综合开发力度，实施北关新城等老城区连片改造，力争全年竣工房地产面积超120万平方米。加快市区工业企业“退二进三”、“退城进园”、机关学校异地扩建进程，以房地产业为龙头，以现代流通业为支撑，大力发展城市服务业，增强城市经济活力。

全面提升城市管理水平。进一步完善“两级政府、三级管理、四级网络”的管理体制，下移管理重心，形成共建共管合力。深化城管执法体制改革，组建城市管理行政执法局，相对集中行政处罚权。高度重视被拆迁居民和被征地农民的生活问题，创新拆迁安置办法。依法组织拆违攻坚，确保城市规划实施。加快“城中村”和小街小巷改造，推动文明创建向背街小巷和城郊结合部延伸。切实加强城市市容环境卫生整治、市政公用设施维修养护、交通秩序综合治理，建立精细化长效管理机制。

加强县城和小城镇建设。按照高起点规划、高标准建设、高效能管理的要求，抓住用好开行贷款，多渠道筹措发展资金，完善县城路网和市政公共设施，加速推进新区建设和老城改造，形成以宿城为中心、四个县城为次中心的发展格局。择优扶持一批中心镇和基础较好的集镇加快建设，搞好规划修编，促进工业向园区集中、人口向城镇集中、土地向大户集中、农业向规模经营集中，进一步提高城镇承载力。

（三）大力发展现代农业，扎实抓好新农村建设。把发展现代农业作为增加农民收入，推动新农村建设的首要任务。加快农业结构调整，大力发展高效农业、生态农业和特色农业，提高农产品优质率和转化率。建立健全畜禽良种繁育体系，做好重大动物疫病防控工作，发展规模化养殖和健康养殖业。围绕粮食、畜牧、水果、棉花、林业、蔬菜六大主导产业，健全“三个一”工作机制，实施“252”强龙工程，发展农产品精深加工。出台优惠扶持政策，建立工作联系和考评制度，精心培育、积极引进、做大做强龙头企业。深入贯彻《农民专业合作社法》，鼓励龙头企业、供销社、种养大户、农技人员领办各类专业合作组织，提高农民组织化程度。完善农产品质量安全体系，畅通鲜活农产品运输绿色通道，推进名优农产品进超市工程。继续抓好农村富余劳动力转移，加强就业培训和输出服务，提高劳动者素质，打造宿州劳务品牌。

加大对农业的投入力度。紧紧抓住国家建设社会主义新农村的政策机遇，积极争取支农项目和资金，加强农田水利基本建设，加大农业综合开发投入。实施农机富民工程，提升农业机械装备水平。加快农村公路、饮水安全和户用沼气工程推进步伐，确保完成1200公里农村公路建设任务，力争用5年时间解决农村人口饮水安全问题，尽快普及农村户用沼气。继续推进农村电网改造，落实城乡用电同网同价政策。加强农村信息服务平台建设。大力发展农村各项社会事业，抓好文化、科技、卫生“三下乡”活动，推动公共服务向农村延伸。切实抓好“十镇百村”示范工程，把村镇规划与主导产业规划结合起来，把点上示范同面上推动结合起来，把政府统筹支持同发挥农民主体作用结合起来，放大示范带动效应，让广大农民群众在新农村建设中得到实惠，树立信心。

全面开展农村综合改革试点。深化乡镇机构改革，建立农村基层工作新机制，提高乡镇政府社会管理和公共服务能力。完善农村义务教育经费保障机制和县乡财政管理体制，增强基层保障能力。稳妥推进土地征用制度改革，建立和完善土地承包经营权流转机制，推进农业规模化经营、集约化生产。积极探索农村合作金融组织创新，组建泗县、灵璧、郊区三家农村合作银行，开展放宽农村地区金融机构准入试点，强化农村金融服务。妥善化解乡村债务，锁定旧债，明确责任，分类处理，防止新债。继续做好第二次农业普查。加强农业执法检查，整顿涉农收费秩序，确保农民负担不反弹。

（四）深入实施东向发展，努力提高对外开放水平。把招商引资作为经济工作的生命线，加快发展外向型经济。准确把握国际国内资本流动和产业转移的新趋势，放大区位、交通、劳动力和资源优势，积极承接资本和产业转移。健全招商引资工作机制，有效整合招商力量，组建专业化招商队伍，推动招商引资工作日常化、专业化、制度化，确保全年实际到位内资80亿元、外资3600万美元。以更大力度优化经济发展环境，强化部门服务企业、服务招商项目的问责，加大投诉案件查处力度，严肃整治行政不作为、乱作为，严厉打击吃拿卡要、公路“三乱”等破坏发展环境、影响宿州声誉的人和事，营造亲商、安商、重商的浓厚氛围。支持优势企业和出口基地开拓国际市场，加强出口商品品牌培育和国际认证，确保全年进出口总额完成4800万美元。强化与徐州经济圈的发展合作，打造与其互动的工业品配套基地、农产品加工供应基地、生态旅游休闲基地。

全力加快开发区建设。市县（区）两级政府必须按照“拉框架、增功能、建项目、树形象”的总体要求，积极创新投入机制，加大对开发区的投入，提升园区基础设施和配套水平，创优局部发展环境，增强各类开发区的自主发展能力。加大开发区招商引资、项目建设和主导产业培育力度，提高项目投资强度和产业关联度，引导园区向专业化、特色化、规模化方向发展，努力把开发区建设成为工业主导型的新城区、特色产业的集聚区和体制创新的先行区。

（五）加大项目建设力度，培植经济发展后劲。把项目建设作为加快发展的关键环节，锲而不舍，强力推进。按照“在建项目抓竣工、新上项目抓进度、前期项目抓开工”的原则，健全项目推进工作机制和管理部门联动机制，强化跟踪考核和督查落实，加快项目实施进度。积极创造条件，加快丰原60万吨农产品加工、华电国际秸秆发电、信诚化工100万吨复合肥、海升6万吨果蔬汁、南虹纺织5万纱锭、中亚博文食品工业园、220KV沱河输变电工程、泗永高速公路和钱营孜、金黄庄、龙王庙煤矿等项目建设。积极做好京沪高速铁路宿州段建设的协调服务，继续推进华电二期、汇源电厂大带小、林浆纸一体化、宿淮铁路、明徐高速、皖北粮食物流中心等项目前期工作，确保年内有实质性突破。针对国家提高项目准入门槛的要求，切实抓好项目的策划和储备，提高项目运作水平和前期工作深度，充实完善“6+3”工程项目库，并争取有更多项目进入国家和省“861”计划盘子。

切实增强消费对经济增长的拉动作用。围绕建设皖东北商贸中心，依托综合交通枢纽的聚合功能，统筹产业布局和市场体系建设，加快城市商业和物流产业发展，积极引进知名流通企业，构建全方位、多层次的大流通格局。充分利用中国宿州灵璧石文化节和中国宿州黄淮海地区农资博览会平台，建设奇石、书画市场，扩展光彩城农资市场，打造宿州会展经济品牌。完成“万村千乡”市场工程布点建设，健全农村商品流通和消费服务体系，大力开拓农村市场。整合全市人文旅游、生态旅游、红色旅游资源，做强做旺旅游产业。制定出台促进服务业发展的政策措施，推动邮电通讯、金融保险、信息咨询及其他新兴服务业快速成长，不断提升服务业的层次和水平。

（六）广泛兴起全民创业热潮，提升县域经济发展水平。把推动全民创业作为奋力崛起的根本途径，完善和落实促进全民创业的政策措施，形成有利于各类市场主体创业的体制机制。大力激活民间资本，凡国家法律法规未禁止的行业和领域，一律向各类创业主体开放。充分激发能人的创业热情，鼓励科技人员、复退军人、高校毕业生、公务员、下岗职工、失地农民开展多形式创业，鼓励外出务工人员“凤还巢”返乡创业，并在企业注册冠名、工商税务登记、经营场所使用等方面，降低准入门槛，给予相关税费减免和资金扶持。健全创业服务体系，加强创业指导和培训，推进创业基地建设。建立民营企业发展的激励机制，开展十强民营企业、十佳服务单位、十大创业能人评比表彰活动，大力宣传创新创业的先进典型，着力营造发展有责、创业光荣的社会氛围。

全面振兴县域经济。落实加快县域经济发展的政策措施，加大对县域经济发展的支持和放权力度，改善县域经济发展条件。鼓励各县区发挥优势，放大特色，突出发展民营经济和外向型经济，积极培育资源型、劳动密集型、农产品深加工型等特色优势产业，全面加快县域工业化、城镇化和农业产业化进程，推动传统县域经济向现代县域经济转型。立足区位、交通、资源、产业等基础条件，积极发展一批沿边型、工业型、旅游型、商贸型的特色城镇，实施乡镇税收增收奖励政策，并在乡镇规划编制、工业功能区建设、重点建设用地、财政性扶持资金安排等方面予以倾斜，培植一批经济强镇，推动县域经济加快发展。

四、以人为本，促进社会和谐发展

建设和谐宿州，是我们加快发展正在实践的重大课题。各级政府要以社会和谐为目标，把社会管理和公共服务放在更加重要的位置，认真解决关系人民生活的突出问题，让人民群众从经济发展中得到更多的实惠，在社会进步中享受更多的成果。

（一）高度关注民生，切实维护群众利益。认真落实促进就业的各项优惠政策，加快推进创业扶持工程，消除“零就业”家庭。建立农村居民最低生活保障制度，提高农村“五保户”供养标准，从人均每年850元提高到1200元；建立城镇未参保集体企业退休人员基本生活保障机制，探索建立城镇居民基本医疗保障制度，力争用三年时间将城镇未参加医保的居民纳入保障范围；提高城乡医疗救助水平，建立健全重大传染病病人医疗救治和生活救助保障机制；推进新型农村合作医疗制度，扎实做好埇桥、泗县、砀山合作医疗工作，并于明年在全市推开；加强农村卫生服务体系建设，改扩建一批乡镇卫生院和村级卫生室；全面实施义务教育经费保障机制改革，从今年起，全部免除城乡义务教育阶段学杂费，继续对农村贫困家庭学生免费提供教科书，并补助寄宿生生活费；加快消除农村中小学危房，用两年时间完成D类危房改造任务；完善农村部分计划生育家庭奖励扶助制度，对农村只有一个子女或两个女孩的计划生育家庭，夫妇年满60周岁以后，按每人每年600元标准发放奖励扶助金，生育独生女的每人再增加120元。通过上述民生工程，惠及更多的城乡居民。

（二）打造平安宿州，保持社会安定有序。始终保持严打高压态势，适时开展专项整治斗争，重点打击邪教和黑恶势力、“两抢一盗”等侵财性、多发性犯罪。深入开展平安乡村建设，完善群防群治网络，切实加强农村社会治安综合治理。强化安全生产基础工作，坚决遏制重特大责任事故发生。加大食品、药品、农资、烟草打假力度，整顿和规范市场秩序。加强公共安全知识普及和应急演练，抓好地震、灾害性天气的预测预报，增强突发性公共事件应急处置能力。健全信访工作责任制，完善经常性的矛盾纠纷排查调处机制，有效预防和妥善处置群体性事件，努力营造稳定安宁的社会环境。

（三）加强社会事业建设，促进经济社会协调发展。建立多元化的科技投入体系，加速科技成果转化，提高技术创新能力。建立健全激发创新活力的人才制度，大力培养、使用和吸引创新型人才。全面实施素质教育，积极发展以就业为导向的各类职业教育，规范发展民办教育；加强薄弱学校建设，优化中小学校布局，不断改善宿州学院办学环境。加快发展医疗卫生事业，启动建设市立医院和皖北煤电总医院门诊大楼，提高医疗卫生条件。健全公共文化服务体系，广泛开展全民健身活动，开工建设市博物馆和体育中心。落实社会事业属地管理制度，加强社区建设。加强精神文明建设，弘扬宿州精神，大力开展社会主义荣辱观教育，努力在全社会形成知荣辱、讲正气、树新风、促和谐的文明风尚。充分发挥广播、电视、报刊和政府网站等媒体作用，加强对外宣传工作，着力营造良好的舆论氛围，提升宿州区域形象。深入实施“五五”普法规划，提高全民法律素质。加强国防教育，抓好人防和国防后备力量建设，积极开展拥军优属、拥政爱民活动，巩固发展军政、军民团结。扎实做好民族宗教工作，充分发挥工会、共青团、妇联等人民团体联系群众、服务群众的作用，切实维护妇女、儿童及农民工合法权益，积极发展慈善、社会福利、残疾人和老龄事业。

（四）促进人与自然和谐，实现可持续发展。继续稳定低生育水平，建立“村为主”长效机制，保持打击“两非”高压态势，努力降低出生人口性别比。切实加强国土、矿产等资源管理，实施城市土地开发利用情况的跟踪监控，坚决遏制圈占土地的投机行为，提高土地集约利用水平，缓解建设用地供需矛盾。加强环境保护和生态建设，实行最严格的环境保护制度，加大生态环境基础设施建设，下大力气解决生活污水、工业废水废气和农村面源污染问题。大力发展循环经济，加强节地、节水、节能、节材和资源综合利用，培育一批循环经济型企业、园区和农业示范基地。

五、与时俱进，切实加强政府自身建设

实现奋力崛起，构建和谐宿州，各级政府肩负着重要职责，必须切实加强自身建设，着力构建法治政府、诚信政府、效能政府和清廉政府，为全面完成今年各项任务提供保障。

（一）加强学习，增强本领。各级政府工作人员要牢固树立学习为本的理念，把加强学习作为创新思路、推动工作的第一需要，加快知识更新，优化知识结构，丰富知识储备。各级领导干部要率先垂范，认真学习现代经济知识、科技知识、社会管理知识和法律知识，紧密联系工作实践，注重对国家宏观政策、经济社会发展重大问题的思考和研究，把学习的体会和成果转化为谋划工作的思路、促进工作的措施，不断提高驾驭市场经济、谋划发展、领导发展的能力，提高推进自主创新的组织能力，提高管理社会事务和依法办事的能力，把精力集中在想干事上，把本领体现在会干事上，把目标锁定在干成事上，创造性地把上级的决策贯彻好、落实好。

（二）转变职能，依法行政。各级政府工作人员要牢固树立管理就是服务的理念，全面贯彻《行政许可法》，加快政府职能转变。深化行政审批制度改革，进一步清理各类行政审批和收费项目，力争使我市行政审批事项在全省最少、收费最低。推进市县区行政服务中心联动，开展相对集中行政审批权试点，推行首席代表制，提高窗口办结率和办结时效。依法完善行政管理决策机制，对涉及经济社会发展全局的重大事项，健全协商协调机制，提升决策科学化、民主化水平。完善政务公开工作机制，拓宽公开领域和渠道，增强政府工作透明度。加强规范性文件的合法性审查，注重通过行政复议等法定渠道预防和化解行政争议。自觉接受人大法律监督和政协民主监督，认真办理人大代表议案、建议和政协委员提案，高度重视人民群众与新闻舆论监督。

（三）改进作风，提升效能。各级政府工作人员要牢固树立脚踏实地、求真务实的工作作风，进一步深化“两风”建设，着力解决少数干部和机关工作人员不思进取、不负责任、不守规矩等突出问题，切实增强各部门服务基层、服务群众、服务市场主体的意识。各级领导干部要立言立行，做真抓实干的表率，敢抓敢管、敢于负责，多解决一些影响改革发展稳定的问题，多办一些为广大群众排忧解难的实事。进一步完善重大事项和重点工作落实机制，加强目标管理，严格责任追究，增强政府执行力，确保政令畅通。着力打造信用宿州，营造“政府诚信、企业守信、人人讲信”的社会信用环境。

（四）从严治政，廉洁从政。各级政府工作人员要牢固树立立党为公、执政为民的执政理念，自觉遵守廉洁从政的各项规定，坚持以人民利益为重，以宿州发展为上，清白清廉做人，公平公正做事。建立健全教育、制度、监督并重的预防和惩治腐败体系，形成用制度规范行政行为、按制度办事、靠制度管人的长效机制，从源头上预防和治理腐败。充分发挥监察、审计等监督机关的作用，加大对各级领导干部和权力部门、重点行业、重点项目和资金的监督力度，严肃查处工程建设、土地出让、产权交易、政府采购等重点领域的商业贿赂案件，从严防范和惩治公务人员职务犯罪行为，以清廉的政风引领社会风气，创造风清气正的社会氛围。

各位代表，放眼宿州大地，600万宿州人民加快发展、创新创业的热情空前高涨；展望美好前景，我们充满着决战决胜的信心与豪情。让我们高举邓小平理论和“三个代表”重要思想伟大旗帜，全面贯彻落实科学发展观，在中共宿州市委的领导下，团结和依靠全市人民，负重前进，奋勇争先，以不断加快发展的新成就迎接党的十七大胜利召开！
